# Supplemental Review of Practical Medicine

**Published:** 1820-06-01

**Authors:** 


### Supplemental Review

OF

### PRACTICAL MEDICINE;

SELECTED AND ARRANGED, WITH COMMENTARIES.

*Paucis libris immorari et innutriri oportet, si velis aliquid trahere, quod in animo fideliter hæreat.* SENECA.

Duo vitia vitanda sunt in cognitionis et scientiæ studio. ***** Alterum est vitium, quod quidam nimis magnam operam conferunt in res obscuras atque difficiles, easdemque non necessarias. CICERO.

*The Supplemental Review in ourlast Number has been so much approved by those on whose judgements we can depend, that we shall continue it, as a useful article, in which we can arrange many subjects that would come in very aukwardly under any other heads.*

### § I. HEAD.

1. *Bony Tumour of the Skull.** We do not recollect any case similar to this on record; but it has greater claims to our attention than its novelty. A young woman, ætat. 18, presented herself to Mr. Keate in March 1815, with a large tumour on the frontal bone, chiefly over the left orbit, and occupying the greater part of the left portion of the frontal bone. It began, six years previously, by a small hard tumour, about the size of a hazel nut, towards the lower part of the bone over the left brow, increasing slowly at first, but during the last three years making a very quick progress. It was, at the date of presentation, the shape and size of three fourths of a large orange, the surface not being quite

* History of a Case of Bony Tumour successfully removed from the Head of a Female. By ROBERT KEATE, Esq. &c. &c. &c. Surgeon to St. George's Hospital. Medico-Chirurgical Transactions, Vol. x.

regular. Latterly, symptoms of internal pressure have come on, as intense head aches, with occasional vertigo, dimness of sight, nausea, and tinnitus aurium.

The tumour was evidently of long growth, and Mr. Keate justly conjectured it was seated between the two tables of the frontal bone; the external table pushed out thereby. She was admitted into St. George's Hospital, where an operation was determined on, and performed in the following manner.

"A crucial incision was made through the integuments covering the whole extent of the tumour; the flaps were turned back, and the bony covering of the tumour itself was exposed; it appeared very thin, and extremely vascular. It had been my intention to remove this protruding portion by sawing through the whole circumference of its base, close to the general surface of the os frontis; accordingly, by means of a large metacarpal saw, about one-third of the circumference was already divided, when, through the groove made by the saw, one of the surgeons who was assisting me thought he perceived a pulsation, as if of the vessels of the dura mater, though this was not observed by myself. I determined, nevertheless, to detach a portion of the bone, in order to ascertain the precise nature of the tumour, before I proceeded further in sawing through the base. This was readily effected by the elevator, when a thin transparent membrane was discovered closely lining the bony case; but in breaking off this small piece of bone, the cyst was ruptured, and its contents, a thin colourless fluid, escaped; the cyst at the same time collapsing into the cavity. This cavity was carefully examined by the finger. It presented an irregular surface, or floor, lined by the membrane above described, but evidently depressed below the general or proper level of the internal table. No pulsation was now perceptible, and no orifice leading through the internal table, and communicating with the meninges, was discoverable. Some more small pieces of bone were then removed, but the patient had by this time become so faint and exhausted, that it was thought prudent to discontinue the operation, hoping that the remainder of the bony case or covering might be subsequently detached, or destroyed by acids or by other means." P. 280.

A little dry lint was applied to the surface of the cavity, and the flaps of the scalp simply laid over it. Considerable reaction followed the operation, but was met by judicious antiphlogistic measures, and ultimately reduced. The operation was performed on the 3d of April. From the 10th to the 20th of that month, pieces of bone were removed from day to day. Subsequently, potential and even the actual cauteries were applied to exuberant granulations and protuberances of an hydatid nature. These hydatid tumours were frequently punctured, and discharged fluid contents. The detail of the subsequent treatment, though interesting, is too long for analysis, and must therefore be passed over. She ultimately recovered, after tedious convalescence, and several inflammatory attacks. The whole case does great credit to the operating surgeon.

---

2. *Ophthalmia.** The Prussian Garrison of Mayence has suffered severely from contagious ophthalmia, the infection being attributed to the communication of the Prussians with the English troops at the battle of Waterloo. When the pain was excessive, they used cold collyria with opium dissolved therein. Blisters, setons, vomits, calomel, gave no decided relief. Excision of the conjunctiva was found more serviceable than scarifications. General blood-letting and leeching were far inferior, in efficacy, to temporal arteriotomy. The Prussian surgeons pinched up a fold of skin on the temple and made a perpendicular incision in it. This being let loose, the artery was easily found, and opened longitudinally with the lancet. A pound and a half, or two pounds of blood were usually drawn, and then the artery was secured by ligature to prevent bleeding or aneurism. This is a preferable mode to that of bandaging, which increases the headache in ophthalmia.

Dr. Kluyskins, who in the years 1817 and 1818, treated, at the military hospital of Ghent, 1870 cases of ophthalmia, considers sanguineous depletion, by temporal arteriotomy, and the application of compresses wet with equal parts of camphor julep, and alkohol, to the eyes, as the basis of the treatment. He abstracts from fifteen to twenty ounces of blood at a time, or until deliquium animi succeeds. In this way he produces speedy relief; and prevents rupture of the cornea, which sometimes took place on the second day of the inflammation. He has renounced the emetic plan of Sir William Adams in general, and only employs vomits where there is evident gastric derangement. For those granulations of the interior of the eyelids which take place in chronic states of the disease, the Prussian surgeons employ the sulphate of zinc, or first a solution of muriate of quicksilver in water, and immediately afterwards, to the same surface, a solution of nitrate of silver, washing both applications off as quickly as possible with cold water. Those periodical pains of the head and eye-balls which are apt to come on towards

---

* Notice sur L'Ophthalmie Epidemique, &c. Journal Gen. de Medicine. Janvrier, 1820.

night, were successfully treated by large doses of cinchona which seemed to have no unfavourable effect on the inflammation that still remained. In very obstinate cases he prescribed with success large doses of calomel and opium.

---

### § II. ABDOMEN.

☞ *Some Sections are occasionally omitted.*

3. *Gastrotomy**. The following case is so well authenticated by three medical gentlemen, as to leave no room for scepticism. It opens a wide field for enterprize in surgery, and evinces the courage of our continental brethren in undertaking daring and dangerous operations to save the lives of their patients.

Madame R——, 29 years of age, had experienced some troublesome accouchemens, after the first of which the abdominal parietes became so relaxed as to hang down on the thighs. In the course of the third pregnancy she had a fall on the left side. After the fifth accouchement she was affected with frequent vomitings, which continued ever afterwards. During this last utero-gestation she perceived a tumour in the left umbilical region, which induced her to think she carried twins. The swelling, however, continued after delivery, and, in fact, increased rapidly, attended with various distressing symptoms, as lancinating pains, indigestion, vomitings, troubled sleep, heat and dryness of the skin, and slow fever. The tumour was moveable, and about four inches in diameter; it could be drawn outwards so far, that the fingers could nearly meet under it, which encouraged the hope that it had no attachment to any of the abdominal viscera below, excepting the epiploon, from which it was believed to originate. The lancinating pains, the hardness of the tumour, and other phenomena, led M. Carré, M. Marechal, and M. Thermonia, of Sedan, to view it in the light of a scirrhous production; and that nothing but its removal could offer a hope (and that they acknowledged to be a very feeble one) of preserving life. Such was the dreadful state of this poor woman's sufferings that she determined on the operation, to the surprise of the surgeons, who little expected that she would consent to such a desperate alternative.

---

* Observation d'une Gastrotomie Pratiquée avec Succés à Sedan; communiquée par M. Marechal, Docteur en Medecine.—Seance du 16 Nov. 1819. Société de Medecine.

On the 1st day of March, M. Carré, assisted by the two gentlemen above mentioned, proceeded to operate. The patient being placed on a table, M. Carré first made a *transverse* incision, seven inches in length, midway between the umbilicus and the cartilago ensiformis, two thirds of which incision was to the left of the linea alba. A second incision descended from the first, along the linea alba, to about four inches below the navel, avoiding the latter by a gentle curve in the direction of the said incision. The angles formed by the two cuts were quickly raised from the anterior face of the tumour, to which, however, the integuments firmly adhered. The dissection was continued, so as to expose the whole of the front, and part of the sides of the swelling. Two needles armed with strong twine were then passed, crosswise, through the morbid mass, and the four cords thus twisted together, gave complete command over the tumour. The abdomen was then opened, of necessity, to detach the tumour, and it was soon perceived that a portion of intestine, and still more of epiploon, were adherent to its internal surface. The intestine was separated by means of the fingers alone, and without much force. The detachment of the epiploon required the knife, and the tumour was at length removed.

At this moment the patient (who had hitherto evinced the most heroic courage) became blanched in countenance, with quick breathing, sense of suffocation, and vomiting, and incessant hiccup. The pulse also disappeared; and, in short, she seemed on the point of expiring. The wound was therefore quickly dressed. The intestines were gently washed with warm water, wine, and oil, the clots of blood carefully removed, and a single suture made at the left angle of the wound. Numerous slips of adhesive plaster, with proper compresses and bandages, secured the whole. The patient was recruited by a little warm wine, placed carefully in bed, with the head and pelvis somewhat elevated, and an anodyne administered.

The tumour was examined, and found to be ten inches long, by seven and a half broad, and six inches in thickness. It was partly of a mamillary kind of substance, partly degenerated into " a putrilaginous matter," (une matiere putrilagineuse) and weighing altogether about seven English pounds.

A long and minute journal is given of the first two months after the operation; but which we think it unnecessary to detail here. A number of alarming symptoms, particularly of the nervous kind, immediately succeeded the operation; and the poor woman was harrassed by great and repeated collections of matter in various parts of the abdominal parietes, that often reduced her to the brink of the grave. She so far recovered, however, as to be able to walk, and ultimately removed into the country among her relations, where, however, she died, not, it is said, of the effects of the operation, three months and six days from the removal of the tumour.

Notwithstanding the success which crowned the operation itself, formidable as it was, the Parisian editor condemns the attempt. We are disposed to look more favourably on the operation, even had it failed in the first instance, which it certainly did not; and we cannot approve of the many "*ifs*" which the critic introduces as objections to the operation of opening into the peritoneal cavity. When we see musket balls penetrate, and traverse the abdomen; when we see sabre wounds exposing the intestines; and when we know that in these accidents, and in embriotomy itself, life has often been preserved, we see no reason why, under certain circumstances, the abdomen may not be laid open, for the removal of morbid growths, as well as morbid collections. We stab a large trochar through the peritoneum in dropsy, without any hazard; but few would venture to assert that the same could be done with similar impunity, if all were sound in that cavity.

P. S. *Another Case of Gastrotomy.** A woman, 24 years of age, wishing to induce vomiting, introduced the handle of a silver fork into her throat, which excited such a strong contraction of the œsophagus, that the fork was drawn out of her hand, and passed into the stomach, where it remained three months, without occasioning any other inconvenience than a sense of weight. The prong end of the fork could be felt about two inches above the umbilicus, the other extremity appeared to be under the liver. After three months had elapsed, however, pain and vomiting, with progressive emaciation, swelling in the gastric region, &c. were developed; and two hundred and twenty-nine days after the accident, M. Cayroche performed the operation of gastrotomy. An incision, two inches in length, over the most prominent part of the tumour laid open the cavity of the abdomen to that extent. The stomach was then opened into, and the fork easily extracted. The wound was properly dressed, no sinister accident occurred, and the patient was perfectly recovered by the twentieth day from the operation, nor has she since that time experienced any inconvenience.

---

* Operation de Gastrotomie; par M. Cayroche, M. D. à Mendes.—Vide Journal General de Medecine for January, 1820.

*Inversion and Extirpation of the Uterus**. Mr. Windsor suspects, perhaps with justice, that inversion of the uterus is probably not so rare an occurrence, even in the present improved state of obstetric practice, as many suppose. As the accident is generally thought to imply a degree either of carelessness or rashness, on the part of the accoucheur in the extraction of the placenta, he will keep it as secret as possible, while an early death often assists in throwing the shade of oblivion over the patients themselves. Mr. W. is acquainted with three or four cases which occurred in his own neighbourhood.

Happily the accident admits of remedy by speedy reduction of the uterus. The hand of the accoucheur should be retained in the cavity of the womb until it has considerably contracted; and the patient should be confined to the recumbent posture for some time afterwards. Our author thinks, that where the uterus and vagina are in a relaxed state, and the woman has been subject to prolapsus uteri, there is a greater disposition towards inversio uteri, during labour, than when such condition of parts does not exist. In such cases the medical attendant should be extremely attentive and cautious in assisting the expulsion of the placenta. It is also highly proper for the accoucheur, after the separation of the secundines, to examine if the os internum be free, while the other hand is placed on the abdomen to ascertain if the uterus be in its natural position.

If such delay have occurred before the practitioner sees the patient, as renders reduction of the inverted uterus impracticable, then a train of symptoms ensue, from the hæmorrhage and unnatural position of the organ, as demands all our care. Some degree of inflammation and fever supervenes, the abdomen becomes full, and tender to the touch, with some degree of hardness at its lower part; there is costiveness of the bowels, and sometimes retention of urine requiring the catheter.

"By the use of fomentations, enemata, laxatives, and an antiphlogistic regimen, the symptoms abate, the power of expelling the urine, especially if the uterus is first raised a little in the vagina, is regained, and the patient gradually recovers the full powers of this function." 362.

Although the patient regains a certain degree of health,

---

* Some Observations on Inversion of the Uterus; with a Case of successful Extirpation of that Organ. By John Windsor, F. L. S. one of the Surgeons to the Manchester Eye Institution.—Medico-Chirurgical Transactions, Vol X. 1819.

yet sanguineous discharges, after a time, return profusely, and her exanguious countenance and emaciated appearance sufficiently indicate the debilitated state of her constitution. After she ceases to suckle, the menses return more regularly; the discharges of blood are more considerable in quantity, and of longer duration; the mucous secretions are copious in the intervals, and the constitution begins to sink under the reiterated losses it sustains.

"In this painful extremity, the extirpation of the uterus itself has been proposed, as the most efficient means of relief; and, formidable as the operation at first view seems, it is known to have been already performed with success."

The public are aware that Mr. Newnham and Dr. Davis have successfully removed the uterus; and our author details the following case, which we shall somewhat condense.

"On the 10th of January, 1817, Mr. W. was requested to visit Harriet Barwick, ætat. 30, who had been delivered of her first child on the morning of the preceding day. The labour had gone on well, and an intelligent surgeon having waited about an hour for the expulsion of the placenta, introduced his finger into the vagina; and finding it, as he thought, descended, he extracted it without any immoderate force. Its removal, however, occasioned excessive suffering to the patient, with violent hæmorrhage, tinnitus aurium, and ultimately syncope. Finding that a descent of the fundus uteri had accompanied the expulsion of the placenta, the accoucheur pushed it up beyond the os uteri. In the evening he examined, and found it not in the vagina; but next morning it descended into that cavity.

"In consultation this evening, they found that the patient had lost much blood; the abdomen was tumid, hard, and tender; pulse 120, and small; no stool since the 6th instant, although castor oil and injections had been exhibited. On introducing two fingers within the vagina, Mr. Windsor felt the inverted uterus, about as large as the fist, increasing somewhat in diameter upwards, and passing through the os uteri, which was in its natural situation, and, of course, considerably dilated. 'It protruded into the vagina nearly to the os internum, felt hard and rugous, and was tender to the touch.' A second attempt at reduction was made in vain. So much inflammatory disposition had now invaded the parts as to render further efforts of this kind not only imprudent but unavailing. Palliative treatment alone was deemed advisable. The abdomen was ordered to be frequently fomented, enemata to be thrown up, salts and senna exhibited."

By these and other judicious measures the inflammatory symptoms were subdued; but no means were efficacious in replacing the uterus. The sanguineous discharges, after weaning the child, were now evidently undermining her health; and finally, on the 22d of August, the operation for removing the uterus was performed.

Mr. Windsor having passed up the fingers of his left hand, unexpectedly found the uterus so relaxed, that he could draw it down, and, without difficulty, he brought it into sight, about two inches beyond the os externum.

"A single ligature of the strongest dentist's silk was thus easily passed round it, and tied as firmly as possible. Beside this, a similar ligature, inclosed in a canula, was passed round the same part, and each end secured to a ring placed on each side of the base of this instrument."

The uterus was now pushed up to the place it previously occupied, and the woman expressed surprise at the operatio being so soon, and so easily accomplished. She suffered little pain till about five minutes after the ligature was applied, when it became severe, and continued so for about half an hour. An anodyne draught was exhibited; and, as she complained of great heat at the lower part of the abdomen, a camphorated spirituous embrocation was applied warm to the parts. Saline effervescing draughts were also exhibited. In the evening the pain diminished; no vomiting; belly soft and easy; slight sanguineous discharge from the vagina. A starch enema, with a drachm of tincture of opium, was injected at bed-time. During the night and succeeding day she had some sleep, with occasional pain, especially in the fore part of the right thigh. The ligature on the canula was tightened about one fourth on the evening of the 23d, which gave a good deal of pain for a short time. The ligature was again tightened the next day; but the pain was of shorter duration. 25th. The ligature being tightened firmly to night, she felt much pain from it. The ligature was now tightened very much daily, in order to deaden the sensibility of the uterus as effectually as possible.

On the 30th, Mr. Windsor again brought down the uterus to view. It appeared rather increased than diminished in size. The ulceration of the ligature had now extended about three quarters through it. A single waxed ligature was again tied *very firmly* round it; the canula ligature was also tightened, and then the uterus replaced.

On the evening of the twelfth day from the operation, it was found that the thin, or rather the broad peritoneal surface of the uterus was the only portion remaining undivided. It was separated therefore by the scissors, which gave her very little pain, and was followed by no hæmorrhage. The remaining cervix uteri was now gently raised, and supported by a sponge and T bandage. On the 11th of October she was walking about, and ultimately recovered perfect health.

We were obliged to pass over the minute but necessary diurnal details, which reflect great credit on the judgment, intrepidity, and unremitting attention of Mr. Windsor. At several periods the patient was in a critical situation, but the rebellious symptoms were always subdued by the exceedingly judicious measures so promptly adopted by the surgeon. Mr. Windsor is entitled to the grateful esteem of his professional brethren, for this additional proof of the great utility of surgery.

---

5. *Suppressed Menstruation.** Nothing can more strongly prove the importance of *regular menstruation* in the preservation of health, than the derangements occasioned by disturbances of that process. The stomach, as it closely sympathises with the uterus, is often the sufferer on these occasions, as the following cases will shew.

A female, 25 years of age, on the third day of menstruation, was obliged to expose herself to wet and cold, while washing clothes at a rivulet, in the month of March. The menses, which usually lasted five or six days, were suddenly suppressed, when she was immediately affected with head-ache, inappetency, lassitude, &c. At the end of three days from this time, she was received into the Hotel Dieu, with the following symptoms. Face flushed; head-ache; furred tongue; pain in the epigastrium; nausea; colic; constipation; hard, quick pulse; restlessness; great lassitude in all the limbs. Twelve leeches were applied to the *pudendum*, the bites of which bled long and copiously. The menses did not return, but the night was passed in tranquillity; and the gastric fever was signally mitigated next morning. In four days she was discharged cured.

This case illustrates the propriety of keeping the *causation* of diseases in view while treating them. It is highly probable that vascular and intestinal depletions would not have been nearly so efficacious or prompt in their effects, as this *local* abstraction of blood from the neighbourhood of the organ which originally sustained the morbid impulse. This *reasoning* is strongly supported by the following *facts*.

A female servant, 22 years of age, experienced, without any ascertainable cause, an interruption of the catamenial discharge for four months. At each epoch of the menstrual

* Dict. des Sciences Med.

revolution, however, she was seized with colicky pains in the bowels,, and inclination to vomit. She was not pregnant. She was received into the HOTEL DIEU in the following state. Flushed face; sub-orbital cephalalgia; inappetency; foul tongue; epigastralgia; nausea; colic; constipation; hard, quick pulse; hot skin; restlessness, and pains in the limbs. An *emetic* of ipecacuan prescribed, which brought off some bilious and mucous matters. In the evening there was jaundice, with an exasperation of the epigastric pain, colic and head-ache. The symptoms continuing unabated, on the second day, fifteen leeches were applied to the *pudendum,* with diluent regimen. Under this treatment the abovementioned symptoms disappeared in a few days, and she was discharged cured.

---

6. *Rupture of the Uterus not Fatal.** No event can be more appalling to the medical practitioner, than rupture of the womb in parturition. Every fact, therefore, which can help to steady his mind upon such trying occasions, it would be culpable in us not to record in a journal of practical medicine.

On the evening of September 1st 1817, Mary Debont, 33 years of age, of weak constitution, and arrived at the full term of her fourth pregnancy, felt the pains of labour. A slight obliquity of the child's head on one side of the superior aperture of the pelvis, offered some resistance to the expulsive efforts of the uterus, enfeebled, probably, by a fall which the woman experienced a fortnight previously. The womb gave way by a transverse rent across its fundus, and the child immediately escaped into the general cavity of the abdomen, where it became prominent, while the uterine tumour disappeared. Gastrotomy was the only resource; but before M. Bernard could procure the assistance of two of his brethren, *twelve hours* elapsed. An incision was then made in the linea alba, beginning two inches below the navel, and continuing it to within two inches of the pubis. The child was dead, of course, and a considerable quantity of blood was found extravasated in the abdomen, mixed with liquor amnii. The intestines were found inflamed, and in some places, discoloured by the long pressure of the fœtus. The

---

* Rupture de Matrice, Suivie, de Guérison. Par M. M. BERNARD, LATOUCHE, et JOSSET. Journ. Comp. Dec. 1819.

extravasated fluids being removed, the lips of the wound were brought in contact, and kept so by sutures. Fomentations, antiseptic drinks, and enemata, and even cordials were given, without any depletion apparently, and the patient recovered in about two months.

---

7. *Nephritis Calculosa.** This case, though sufficiently interesting in itself, is related in a style too colloquial and diffuse for a periodical repository, where every degree of conciseness, consistent with perspicuity, should be a prominent feature. It is our duty, however, to remedy, in some measure, the evil, and illustrate the precept by example.

The patient, about 40 years of age, of liberal education, cheerful disposition, and unconquerable firmness of mind, divides his illness into four periods. In the first period, commencing in 1801, he began to discharge calculous matter, while otherwise in perfect health, at first with little pain, and no particular sensations in the region of the kidnies. The calcareous discharge was a white chalky substance, passed immediately on voiding his urine, and enveloped in thick mucus. In 1802 the discharge became more constant, and attended with some irritation at the neck of the bladder and along the urethra, the mucus being sometimes tinged with blood. The calculous substance was now in the form of a pretty stiff paste, which hardened on standing, crumbled between the fingers, and exhibited sparkling crystalline particles. The patient was now about 23 years of age, and active. Towards the close of 1803, his habits became sedentary, and he now experienced violent paroxysms of pain in the lumbar region, attended by retchings and constipation. Cathartics could only be retained by combination with opium; but whenever they operated, the nephritic symptoms were considerably relieved.

In 1806-7, these attacks became less frequent, which the patient ascribed to less arduous duties, and more exercise. In November 1810, they ceased altogether; but the patient was, soon afterwards, assailed with a different and more formidable train of symptoms—those attending a stone in the bladder, viz. irritation at the neck of the bladder; tormenting itching and shooting pains at the extremity of the ure-

---

* History of a Case of Nephritis Calculosa, in which the various Periods and Symptoms of the Disease are strikingly illustrated. By Alexander Marcet, M. D. F. R. S. Medico-Chirurgical Transactions, Vol. x.

thra; violent nocturnal chordee; difficulty of passing urine, which was frequently tinged with blood. These symptoms gradually increased to the utmost pitch of severity. Dr. Marcet here introduces the patient's own description of these sufferings, (though he acknowledges that they are familiar to the ears of physicians and surgeons) dressed in language considerably tinctured with ultra-minute, and exaggerated representation. This we shall entirely pass over, as quite unnecessary in a purely professional work.

In 1811 he introduced a bougie himself, and distinctly felt it graze upon a hard substance. A consultation was held, an operation determined on, and Mr. Cline performed it. Here the patient is allowed to describe his feelings during the operation; which, being more afflictive than instructive, we shall pass over. The recovery from the operation was rapid, and the patient was soon perfectly restored; in which happy state he remained about eighteen months, when he was suddenly seized with acute pain in the region of the left kidney, which lasted for several hours, when a sharp knock at the street door made him give a sudden start, and the pain instantly ceased. A small calculus was now discharged by the urethra. In a few weeks after this, his urine became habitually tinged with blood, but without pain or "coaguli," [coagula] except in the urine first discharged in the morning. He continued to be subject to dreadful nephritic pains, ending in the discharge of small calculi. Between the attacks he recovered completely, and was not sensible of any affection of the kidney, except a sense of fulness and tenderness in that organ. He also continued free from symptoms of calculus or disease of the bladder.

In 1816, when Dr. Marcet was first consulted, the calculus extracted by Mr. Cline was examined, and found to be of the fusible kind, as were also the fragments subsequently discharged. Dr. M. advised the patient to discontinue all alkaline medicines, and take small doses of the muriatic acid, from ten to fifteen drops, twice or thrice a day, diluted in water. He persevered in this plan nearly a year, the medicine agreeing with his stomach, and seeming to have the effect of diminishing the discharge of mucus, though the habitual hæmorrhage still continued. In January, 1817, however, he had a sharp nephritic attack, which terminated, as usual, in the discharge of a small calculus, and suspended, for a short time, the hæmorrhage. In the July following, he had a long and severe fever, which lasted several weeks, but was apparently unconnected with the calculous complaint. On the third day of this fever the hæmorrhage ceased, and never since returned. The fever was also followed by a long truce of the nephritic attacks. In April last he experienced a paroxysm, and discharged a small stone. He is now in good health and spirits. During the exhibition of the mineral acid in 1817, the small calculi passed continued to consist of phosphate of lime; but Dr. M. could no longer detect any magnesia in them, and they had lost the fusible character. During the year 1818, and some time after the acid had been left off, the magnesia re-appeared in the calculous fragments.

We are not aware of having omitted any fact worth recording in this analysis of Dr. Marcet's paper; and if so, we have caused it to occupy a space in the annals of medicine, nearly seven times less than it originally required. Aussi va le monde! While some are employed in expanding things to the utmost possible dimensions, others are engaged in the work of condensing them back again to natural, useful, and portable shapes.

### § III. NERVOUS SYSTEM.

8. *Tic Douloureux.** Almost the whole of our life is taken up in the pursuit of pleasure, or the evasion of pain. The former is but a fleeting enjoyment, while the latter is the common and constant attendant on humanity, from the cradle to the grave! Those silk-like cords which convey sensation to the brain, and volition from that organ to the muscles or other parts, evince no cognizable change or motion, in the performance of these two opposite functions. They may also be thrilled with exquisite pleasure, or tortured with excruciating pain, and still exhibit no visible indication of either, in their structure. In this state of our physiological and pathological darkness, then, respecting the nervous system, we must explore our way by the aid of such facts as an empirical practice shall ascertain; for we have few or no general principles to guide us through this labyrinth of uncertainty. The author of the work before us has added another remedy—namely, IRON, to the list of those which have been tried, and too often unsuccessfully, against tic douloureux. His motto is appropriate-

* Cases of Tic Douloureux successfully treated. By BENJAMIN HUTCHINSON, Member of the Royal College of Surgeons, &c. &c. Octavo, pp. 71. London, 1820.

ly selected from the promise of Vulcan to Venus, in the 8th Book of the Æneid—

> Quicquid in arte meâ, possum promittere curæ,
> Quod fieri FERRO, liquidoque potest electro.

And if the promise here made, or the hope here inspired, be but even partially realized by the event, Mr. Hutchinson will be entitled to the lasting gratitude of the profession, as well as of his fellow creatures in general. Pain is a greater evil than death, which is merely a negation both of pain and pleasure; and consequently, he who frees a human being from extraordinary suffering, is much more entitled to the civic crown, than he who merely saves the life of a citizen.

Mr. Hutchinson presents himself before the public in a garb of modesty which cannot fail to make a favourable impression on every man, whose approbation it is desirable to obtain. He comes forward with "neither a new remedy nor a new theory;" but merely with "a few observations which he has made on the successful administration of a mineral substance well known, but too much neglected, or inaccurately, or inefficiently employed." Our author does not neglect general means, when circumstances require them; but he attributes his success in the management of a hitherto most obstinate disease, to the activity with which he has employed a remedy formerly administered in doses far too small to produce the desired effects, in this and some other diseases.

The seat of tic douloureux is known to be usually confined to the superior maxillary nerve, or the second of the fifth, and portio dura of the auditory, or pes anserinus distributed over the face. Certainly, however, it occasionally affects other and distant nerves.

"The complaint commences with slight and almost imperceptible attacks of pain, and generally without any warning, though some patients feel in the affected part, peculiar and inexplicable sensations preceding its approach, from which they can announce with horror the coming enemy; the patient at the same time enjoying a good or an indifferent state of health. The pain, however, soon becomes more acute and lancinating, shooting and darting along the various ramifications of the affected nerves; it generally continues from a quarter to half a minute, and seldom exceeds the space of one minute. It returns at intervals more or less frequent; there being sometimes several paroxysms in a few minutes; and at other times there are intervals of from fifteen to thirty minutes, or longer. There is no determinate period; we always find the utmost irregularity, even in the same patient.

"The pains vary in their degree of intensity, at one time exciting the most piercing cries, and distracted writhings and motions in the afflicted patient, while at another they are more bearable. When at the acmé of their violence, the parts affected are often convulsed, and sometimes various contortions and grimaces are observable. These are to be distinguished from the convulsive twitchings of the muscles with which the diseased nerves communicate, and which are occasioned by irritation from the excessive pain; while the contortions and grimaces are voluntary, being caused by the patient's writhing and twisting from the agony of his torture, and may be prevented by a firm resolution to resist any impulse of shrinking from the attack." 13, 14.

The pain does not always confine itself to the seat of the disease, but darts rapidly to the neighbouring parts, like radii from a centre. It rarely gives warning of its approach; and frequently the first sign of an attack is the patient starting up in a state little short of phrenzy. In this condition, some patients beat the parts with violence, or forcibly rub them with some rough substance, till excoriation takes place, by which they sometimes succeed in diminishing the intensity of the pain. The pains are more frequent during the day than in the night, and during conversation than in silence; but most of all at the time of mastication, when the attacks often succeed each other with such rapidity, as to appear like one continued paroxysm. The eye, at times, is red and watery, as in severe odontalgia; in other cases it is particularly dry, and in some patients a copious flow of saliva succeeds the paroxysm.

"When the disease continues for a great length of time with increasing violence, the patient can neither obtain rest by night nor by day; his appetite fails, and, as may be expected, there is some degree of pyrexia: this, however, but rarely happens, and only in cases of the utmost severity.

"Pains, said to resemble those of the morbus crucians, are sometimes met with in other parts of the body. Mr. Cooper mentions a case, in which the radial nerve was affected; and Lentin one, where the pain was seated in the calf of the right leg. If one nerve be affected, surely any other may be equally susceptible of a similar affection." 19.

We shall not dwell on the various histories of cases scattered through periodical Journals, most of which were unsuccessfully treated, even when the nerve was severed by the knife; but proceed at once to the more gratifying part of the pamphlet before us.

The failure of the remedies usually employed to subdue the torments of tic douloureux, induced our author to try the different preparations of Iron, and his investigations were attended with happy results. The preparation of this mineral which Mr. H. prefers, after a fair trial of all its forms, is the ferri carbonas of the London Pharmacopœia. It is prepared by mixing, in certain proportions, solutions of the sulphate of iron and of the carbonate of soda together, when an immediate mutual decomposition takes place. Sulphate of soda is formed, which remains in solution, and carbonate of iron, which is precipitated of a green colour. The precipitate, when first formed, is the carbonate of the black oxide of iron, or contains the iron in the state of black oxide, the state in which it exists in the green sulphate of iron; but in the process of drying, it absorbs more oxigen, becomes of a red colour, and is converted into the carbonate of the red oxide of iron.

"This is the preparation of iron, which, though in general use, has been hitherto very inefficiently administered; in doses so minute as to preclude a possibility of much good effect being produced. I mean not to confine this observation to the management of the tic douloureux, but to extend it to every case in which it is imagined that iron is to be useful. In proper and efficient doses I hope to be able to demonstrate its valuable and highly curative powers." 40.

Mr. Hutchinson has selected six cases from his notes, to illustrate the treatment by iron, and of these we shall present a brief analysis.

*Case* 1. This is detailed in a series of letters from the patient herself, Jane Smith, of Aldercar Park; her complaint was seated in the nerves over the os malæ, just below the orbit, in the gums, teeth, upper lip, and ala nasi; it proceeded therefore from the second branch of the fifth pair of nerves. Various remedies had been tried in vain, before Mr. H. was consulted. The patient had been subject to the tortures of tic douloureux for twenty years, with very little respite. Mr. H. prescribed ferri carbonatis ʒj bis die, ex melle, vel theriaca, which was continued for years, with some intervals, and with complete success.

*Case* 2. This is also related in the words of the patient, Henry Jervis, Esq. of Cheswardine, Shropshire. The delineation is somewhat metaphorical, in the military style of description, a passage or two of which we shall here introduce.

"It sometimes commences with a slight coruscation or ticking, somewhat similar to that of a pendulum, whence it may probably derive its name, being a disease more known in France than in England. It is afterwards succeeded by a shock more violent than that of an electrical machine, but of much longer duration. A red-hot salamander laid upon the head, may afford some resemblance of the effect it sometimes produces. At other times, it may convey some idea of the operation of an incision knife, or tomahawk, the lancets of a cupping instrument being nothing, compared to it. Sometimes you may imagine minute guns passing through the head for a considerable length of time; the patient may at others, suppose his head to be laid open with a battle-axe, and the brain exposed to a dreadful north-eastern blast.

"The first mouthful of meat or drink at breakfast or dinner, often produces some one or other of the above sensations.

"After all, the most extraordinary trait in the character of this disorder is, that after its most violent attacks, the storm subsides as suddenly as it commenced, without leaving a trace behind." 51.

This gentleman took the carbonate of iron in drachm doses twice a day, during a long period, and with great effect. Slight returns of the pain are always counteracted by a fresh recourse to the remedy.

*Case* 3. Mrs. Rayner, ætat. 57, after having borne many children, began to complain of rheumatic pains in the right side of her face, which she attributed to carious teeth, eight or ten of which were extracted, without affording the expected relief. The pains were confined, in the first instance, to the os malæ, the upper lip, and the tongue; the eye, on that side, occasionally shedding a tear from the agony of her suffering; and the upper edge of the temporal muscle suffering repeated contortions. Slightly touching the skin, masticating, or speaking, would immediately excite the pain, "which continuing perhaps half a minute, would suddenly vanish and leave the patient perfectly well." In this state of affairs calomel, opium, and hemlock, were exhibited every eighth hour, with sensible diminution of the complaint. After persevering in this plan for fifteen days, a profuse ptyalism came on; when "the pains gradually abated, and at the conclusion of the salivation, which was very troublesome, the disease appeared to be altogether subdued."

In this pleasing change of health the patient continued for seven or eight months, when, on the sudden accession of severe hepatitis, the excruciating tortures in her face returned with redoubled force. The mercury and opium were again tried, but not with such decided effects as before. The arsenical solution was then pushed to a considerable extent; yet the remedy appeared to possess no authority over the disease.

"In this unpleasant state of things, I had recourse to the ferri carbonas, half a drachm of which I began to administer three times a day, mixed in honey. During the first seven days, but little benefit was perceptible. I then increased the dose to one drachm twice a day, and after the first three days of this increased quantity, a very sensible abatement of the number and violence of the paroxysms was to be discerned. I now increased the dose of the carbonate to four scruples twice a day, in which she regularly persevered for ten weeks, at the expiration of which time she was wholly free from the slightest vestige of this disease, and never afterwards experienced the most trivial return." 55, 56.

For some time afterwards Mrs. R. took what she called a fortnight's course of her brown powder, twice a year, to which she attributed her freedom from assaults.

*Case* 4. Mr. Hage, of Upton, 58 years of age, after an attack of acute rheumatism, complained of agonising electric shocks extending from the inner canthus of the right eye, and also from the globe itself, down the os malæ to the upper lip, the ala nasi, the teeth, and the gums. He suffered also an effusion of tears, which excoriated his cheek. These excruciating tortures, with the exception of the intermissions usually accompanying the tic douloureux, continued with unabated violence for two months, under the exhibition of various measures. Conceiving that the disease arose from carious teeth, he had some extracted, but without any permanent relief.

" In this unhappy state of the disease, I began to try the effects of the ferri carbonas in doses of one drachm twice a day: this plan was persevered in for sixteen days, without much alleviation. I then augmented the dose of the carbonate to four scruples twice a day. This increased dose produced a check upon the violence of the disease: after having used it for five days, the pains, twitchings and contortions began sensibly to abate in duration and in violence, and in the course of a fortnight were apparently removed." 58, 59.

*Case* 5. Mrs. Jane Brown, became affected with tic douloureux when about 22 years of age. The disease was ushered in with symptoms of active inflammation in the adjacent parts, great pulsation of the facial arteries, and considerable febrile irritation. The inflammatory excitement was subdued by proper means, without mitigating, in any material degree, the excess of her sufferings.

" The portio dura of the seventh pair of nerves, spreading its branches to most parts of the face, and communicating with several of those of the fifth pair, distributed to the lower jaw, the mastoid process, and the ear, being here affected, conspired to afflict my patient. She first experienced those peculiar and inexplicable sensations, which usually precede an approaching paroxysm: the pains would then assume their most acute and lancinating character, darting and shooting along the course of the affected nerves: the periods of their duration varied considerably. The pain did not always confine itself to the seat of the disease; but, as I have before observed, it darted with the velocity of lightning to the neighbouring parts, in various directions." 64, 65.

Mr. Hutchinson began by exhibiting half a drachm of the ferri carbonas twice a day, which plan was persevered in for the space of three weeks, without any considerable abatement of the pain. Calomel and conium were next tried, but ineffectually, and the same with the arsenical solution, sulphate of zinc, nitrate of silver, and extract of henbane. Again our author prevailed upon the patient to have recourse to the iron. He began by giving four scruples twice a day. On the fifth day a very perceptible amendment was evident, and a regular perseverance in similar doses rewarded the patient with a gradual extinction of the malady.

We have now laid the substance of the pamphlet before our readers, and shall leave them to judge for themselves, as to the disease and the remedy here proposed. We believe the etiology of tic douloureux to be too varied, for a single remedy to apply in all cases. We think it probable, however, that iron, in large doses, may be effectual in certain species of the complaint. On this account, Mr. Hutchinson is entitled to the respectful consideration of his professional brethren, for this contribution to our therapeutical stock of knowledge.

### § IV. VASCULAR SYSTEM.

9. *Transfusion of Blood.** The pathology of the fluids is a great deal too much neglected in modern times; and this is so clearly seen by some eminent physicians of the present day, that we anticipate a return of attention to this interesting subject. Dr. Blundell is entitled to the esteem of his brethren for his laudable exertions in the advancement of physiological as well as pathological science. May the ardour of youth continue, and be united with the wisdom of age!

In the spring of 1818, Dr. Blundell ventured to recommend the transfusion of blood, in cases of desperate inanition. The experiment has since been tried, and the following is a brief outline of the result. A poor man laboured under scirrhosity of the pylorus, as was ultimately proved by dissection. For some months previously to his becoming the

* Some Account of a Case of obstinate Vomiting, in which an Attempt was made to prolong life by the injection of blood into the Veins. By James Blundell, M. D. Lecturer on Physiology and Midwifery, in Conjunction with Dr. Haighton, at Guy's Hospital.— Medico-Chirurgical Transactions.

subject of the experiment in question, he had vomited the greater part of his food; his bowels were constipated; yet there was no pain, nor tenderness, nor enlargement, in the region of the stomach. Neither did the matters ejected from that organ exhibit any appearance indicative of ulceration. On the whole, there was some little ground for hope that the symptoms might not depend on pyloric scirrhus. The patient, however, was so completely exhausted from defect of sanguification, that his dissolution was hourly expected. Transfusion being determined on in a consultation, it was performed, in the presence of Drs. Cholmely, Back, and Wright, Messrs. South, Callaway, Cox, and Pollard, in the following manner:—

"For this purpose, about an inch of the right cephalic vein was laid bare, a little above the elbow, (for the vessels were too much contracted to admit of the operation below it), and a longitudinal incision, about a line in length, was made with the lancet. Some gentlemen present, undertaking to supply a few ounces of blood, about an ounce and a half was taken up by the syringe, and immediately infused into the vein in a gradual stream. This operation was repeated ten times, so that between twelve and fourteen ounces of blood were introduced in this manner, in the course of thirty or forty minutes.

"No very obvious changes, either morbid or of a salutary nature, made their appearance during the operation. The brain, nerves, and muscles, remained undisturbed; the respiration continued unaltered; the temperature of the body scarcely rose; and even the pulse, with the exception of a slight increase in its size, and a dubious variation of three or four beats in the minute, underwent no obvious change. It should be observed, however, that the livid discolouration of the hands already described, gave way to a more healthy complexion; the same change, though unattended to, probably taking place on other parts of the skin. In reply to repeated inquiries, the patient himself declared, that he perceived no unusual sensation whatever; and at the close of the operation, when speaking doubtfully of his improvment, he expressed himself in a more audible whisper than he had made use of before." 300.

It may be proper to observe, that the different portions of blood were not injected in immediate succession, but at irregular intervals of five or six minutes, so as to give time for each portion to be distributed over the vascular system, before a fresh supply was poured in. The little pipe, easily introduced, was secured in the vessel, without the assistance of a ligature, merely by the pressure of the finger.

The operation, which was performed about three o'clock, produced little effect at the time; "but in the course of the evening, the patient experienced a salutary change from it." His body became warmer; his respiration remained regular; his pulse acquired double its former size, and was 88 in the minute. These favourable symptoms continued all the night, and a great part of the next day, when there appeared a degree of excitement, his limbs being very warm, and a return of appetite evinced.

The next evening (27th September) he began to droop, and sank so rapidly, that on the following morning, he was reduced as low as before the operation. He died at eleven o'clock on the evening of the 28th, about fifty-six hours after the transfusion.

On dissection, it was found that the pylorus was scirrhous, together with the upper part of the duodenum, the indurated mass having made a slight pressure on the gall ducts. There was no ulceration, though considerable stricture of the pyloric orifice.

It need not be observed, that the subject of the experiment was the most unfavourable that could possibly exist. The case, however, proves that there is no *immediate* danger from the transfusion of human blood; and that in desperate cases of inanition, caused by accidental losses, not organic obstructions, the trial of transfusion is completely justifiable.

If a true idea of a man's mind can be gathered from his writings, (which we believe may sometimes be the case) we should be much inclined to augur well both of the head and heart of our author. We think, however, that he indulges too much a disposition to prolixity. He has armed himself, indeed, against this objection, by a sentiment from the great Roman Satirist——

> "Nulla unquam de vitâ hominum cunctatio longa."

To the precept of the Poet, we would oppose the sober sentiment of Hippocrates.

> —"Vita brevis, ars longa, tempus præceps, experimentum periculosum, judicium difficile."

---

### § V. ABSORBENT SYSTEM.

10. *Scrofula.** Pursuant to our plan of leaving theoretical and physiological speculations to others, we shall only present the reader with some account of the practical portion of

---

* A Treatise on the Nature of Scrofula, &c. &c. &c. By WILLIAM FARR, Esq. Surgeon. One vol. 8vo. pp. 76. London, 1820.

this volume. After having discussed the general causes of scrofula, our author comes to the plan of treatment, which he says he has found " pre-eminently successful, both in arresting the further progress, and effectually eradicating a disease so destructive of human life, and inimical to the peace of numberless families." He does not mean to say that he has had this success in " the more protracted forms of visceral disease," or in " tubercular phthisis."

" I mean to assert, (says Mr. Farr) its efficacy more particularly then, as regards certain morbid *changes acting* upon the salivary and contiguous glands, characterized by a *rocky* or irregular surface, with abscess, or making *its* (morbid changes ?) appearance *by* (in) chronic inflammations, and thickening of ligaments and periosteum, with caries of bones." 35.

We have marked some expressions and words in the foregoing extract, to remind medical authors of their too common inattention to language and composition; by which inattention, our writings are proverbially uncouth. The remedies then for scrofula, are Brandish's liquor potassæ, with mercurial frictions. The reasons why the profession were not so successful with the liquor potassæ as Mr. Brandish was, our author undertakes to explain. One reason, he thinks, is, the want of attention to its slow operation; and also, " the almost invariable custom of prescribing the liquor potassæ of the London Pharmacopœia, as a substitute for the caustic alkali, without taking into consideration that the one is possessed of much more powerful alkaline properties than the other." Mr. F. asserts that Brandish's medicine weighs two ounces in the pound (we think he should have said two ounces in the pint) more than the common liquor potassæ. Brandish's liquor also, is given in mean doses of one to three drachms, whereas the liquor potassæ of the shops is rarely prescribed in doses exceeding half a drachm or a drachm. Besides these ponderable differences, Mr. Farr thinks there are medicinal differences, although not discoverable by analysis, but only perceptible in their relative effects on the human body. Our author gives a decided preference to mercurial frictions over an alterative course of mercury internally exhibited. In the former way, he imagines that the medicine exerts a power " upon the whole superficies of the body, by continuity of surface, though its application and friction is (are) limited to a small space, an arm or leg, for example." This power is the removal of any " obstruction to the free exit of the perspirable matter, by increasing the action of the capillary vessels of the skin universally, thus preventing any inordinate accumulation of morbid secretions in the intestinal canal."

In respect to the *alterative* treatment, our author states his belief that mercury, in this way, can only be useful "by exciting the action of the liver, thereby inducing a healthy secretion of bile, thus regulating the action of the bowels, and controlling the various secretions."

The preparation which Mr. Farr employs, differs very little from that of Mr. Brandish. He prescribes it twice a day, between breakfast and dinner, and at night going to bed, in any agreeable vehicle not capable of chemically altering the alkali.

"To children, from four to six years old, I generally give one drachm by measure; from six to eight, one drachm and a half; from eight to fifteen, two drachms; and in some few instances even more. I am warranted by experience to affirm, that no symptoms denoting qualities hurtful to the system, either by excoriation of the mouth and fauces, arising from its action as a caustic, or pain in the stomach after it has been taken, denoting similar effects upon that organ, have ever been observed by me."

In respect to the mercurial frictions, we shall introduce the author's own directions on that head.

"For children, from four to eight years old, I direct five grains of the *unguentum hydrargyri fortius* of the London Pharmacopœia; from eight to twelve, eight grains; from twelve to fifteen, twelve grains; and from sixteen and upwards, from twelve to fifteen grains; which is to be rubbed in every night before going to bed; the friction to be continued until no portion of ointment can be observed to stain a clean finger when applied to the part on which such friction has been employed. I am accustomed to direct the patient to wear a linen glove on the hand which has been used in rubbing in the ointment; which, of course, should be washed off in the morning with warm water, and the arm or leg upon which the friction is made (for it is immaterial), should also be covered with a glove or stocking; but there is no absolute necessity for washing the parts rubbed, oftener than every third or fourth day; and here I would urge the propriety of the patient washing himself always during the use of the mercurial ointment, in water rather warm. In order to prevent the slightest induction of mercurial action in the system, I occasionally administer an aperient draught, composed of a solution of neutral salts, or a small quantity of rhubarb in powder." 47, 48.

Upon regimen, clothing, &c. Mr. Farr makes some observations, together with remarks on the local treatment of scrofulous swellings and abscesses. Our confidence in any peculiar plan of treatment in this obstinate disease, is not great; yet we think that Mr. Farr's methodus medendi is not undeserving of consideration, where so many other means have failed.

11. *Lymphatic Glands.** The disease in question, Dr. C. thinks, should be discriminated from a venereal or scrofulous affection, with which it is generally confounded. Its seat is commonly in one of the lymphatic glands of the lower or femoral range—sometimes, however, in the upper or inguinal row. When about the size of a walnut, the integuments retain their natural colour, and a slight pain only is complained of. The progress of the tumour to suppuration is slow, yet uniform. The matter is tolerably good, but the openings by which it escapes, preserve rather the appearance of fistulous orifices, healing in two or three months, but sometimes remaining open for the space of a year. A very striking feature of this disease is the trifling degree of pain which attends it.

From the earliest period at which our author had an opportunity of observing this complaint, the constitution was found engaged. Head-ache, languor, and a quick pulse, (generally from 100 to 120) accompany the local affection, while all the other functions appear to be well carried on. The patients generally conceived that their health was improved by this disease; for before the final healing of the ulcerated opening, they have informed Dr. C. that they felt themselves in better health than they had enjoyed for some months previous to the attack.

In the treatment, Dr. Colles confined himself to the promotion of suppuration, and to the relieving the head-aches by purgatives. He thinks mercury, in such cases, would be injurious.

### § VI. GENERAL SYSTEM.

12. *Post Mortem Changes.*† It is necessary, in this world, to be as vigilant in detecting error, as zealous in the investigation of truth. In medical science, this maxim is peculiarly applicable to all our pursuits, to all our studies, whether of the phenomena during life, or the appearances after death.

Dr. Davy, in a Letter to Sir James M'Grigor, draws the attention of the Faculty to certain deceptive changes which the human frame undergoes, shortly after vitality ceases, in

* On a Disease of the Lymphatic Glands of the Groin, attended with peculiar Symptoms. By A. Colles, M. D.—Dublin Hospital Reports, Vol. II.

† Observations on the Changes which the Animal Body undergoes in a hot Climate soon after Death. By John Davy, M. D. F. R. S.—Medico-Chirurgical Transactions, vol. x.

a tropical climate—and, perhaps, with some modifications, in all countries. He observes that, "immediately after death, before the body has lost its heat and flexibility, the heart and arteries, as well as veins, are full of blood, which is fluid, as in life, and every part may be considered nearly in the same state that it was in before the fatal event took place."

We confess that this passage somewhat surprised us, since it does not quite accord with the result of our own observations. We have been in situations, both in cold and warm climates, where we had opportunities of examining bodies in the course of one, two, and three hours after death, and long before they had lost their heat and flexibility; yet we could not find this even distribution of blood throughout the arterial and venous systems, which Dr. Davy describes. Every one knows that the blood instantly forsakes the great circle of capillaries on the whole surface of the body, when life ceases; and that the internal trunks, especially of the venous system, become proportionally gorged. How then can every part be considered nearly in the same state as during life?

"If (says Dr. Davy) several hours be allowed to elapse, before the examination of the body is made, for instance, from twelve to sixteen hours, new appearances will present themselves. Little blood will be found in the great arteries;* coagulated blood, or polypi, will be found in the auricles of the heart. The viscera will appear more or less turgid with blood, especially the lungs and the lower part of the viscera; the peritoneal covering of the gall-bladder, and the adjacent parts of the liver and intestines, will have assumed a dark greenish hue: and lastly, should there be much bile in the intestines, the intestines in general, and peritonæum, will be discoloured, and rendered of a light green." 90.

If the interval be still longer, the serous and mucous membranes, in general, will appear red and inflamed, especially in those parts most exposed to the action of the blood, as the valves and lining membrane of the heart and arteries. The serous effusions into the cerebral or pectoral cavities will be more or less tinged with blood — the viscera will appear dark and livid — and the skin along the track of the great vessels streaked of different hues from extravasated blood.

Dr. Davy observes, that it is a well known fact, that the blood, soon after death, and before it has time to coagulate,

---

* We have found the arterial system more or less freed of blood from the moment of death; and unless where local engorgement or inflammation existed, we have observed that it became generally emptied in a much shorter space of time than Dr. Davy mentions. Rev.

partially forsakes the great vessels and accumulates in the viscera. It is equally well known that bile, immediately after death, begins to exude through the gall-bladder, and stain the contiguous parts. The same, he thinks, takes place when bile is plentiful in the intestines.

"The appearances of inflammation, in consequence of the exudation of bloody serum, are the only phenomena which I have described that have any pretension to novelty."

These false appearances, Dr. Davy avers, are so exactly like the true, in many instances, that he doubts if the most experienced anatomist would be able to distinguish between them.

"I have made the trial with medical friends, by asking their opinion of parts that had been coloured by immersion in blood, and without the least hesitation, they have pronounced the parts strongly inflamed."

Upon the above passages, we would remark, that every man, in the habit of opening bodies, and prosecuting pathological researches, has observed the tinges of colour, described by Dr. Davy, on the surfaces of organs in contiguity in the dead body; and even engorgements of the structure of certain tissues and parts, produced by the effusion and gravitation of blood and other fluids, after vitality had ceased—and that without attributing them to inflammation. When parts have been actually inflamed, however, there are, generally speaking, an increased vascularity and thickening of the tissues, which distinguish them from mere *post mortem* infiltrations and discolorations. But we are to bear in mind, at the same time, that inflammation, of the most intense degree, so as to terminate life in a few hours, may exist in the serous tissues particularly, and yet leave no redness or discolouration behind.

"Quelquefois (says Bricheteau) des phlegmasies tres intenses du systême sereux ne laissent, aprês la mort, aucune trace de leur existence; il est probable qu'il y a, dans se cas, resorption du fluide épanché par l'extrême irritation qui a causé une mort inopinée."

In certain varieties of inflammation in the serous membranes, as the peritoneum, pleura, and pericardium, we find (says the same author) only a kind of dryness and roughness of the inflamed tissues. A case is related by Marandel, illustrative of this fact.

"A butcher, (says he) 30 years of age, and of robust constitution, was seized with violent pain in the side, at nine o'clock in the morning, and carried to the Hotel-Dieu one hour afterwards. Every means for reducing pulmonic inflammation was put in force, but he expired at six o'clock the same evening. On strict examination, only a slight blush could be perceived on the pleura pulmonalis of the affected side, but without any effusion or other change of structure." "We have seen (continues Marandel) inflammations of the pericardium produce similar fatal effects in a most rapid manner; and where dissection disclosed no other appearances than a dryness and faint redness of that portion of the pericardium reflected over the heart." "*A l'ouverture des cadavres, on ne trouvait que la secheresse de sa surface, et une rougeur peu marquée sur la portion de cette membrane qui recouvre le cœur.*"

These facts, together with the observations of Dr. Davy, may convince us that *post mortem* appearances must always be studied, in conjunction with the symptoms during life; otherwise, we shall be perpetually erring and drawing false conclusions.

---

### § VII. MEDICINAL AGENTS.

13. *Military Hygiene.** It is the prerogative of *reasoning* man to wage war on his own species; while *instinctive* animals, even of the most ferocious orders, spare their kind, and destroy their weaker neighbours only to preserve their own existence. As three or four thousand years of progressive civilization have not, in the least, diminished the propensity to war, we may fairly conclude, that it is one of those hereditary evils permitted to cling to our nature by a wise Providence, and for necessary, though inscrutable purposes. The fiery hurricane of WATERLOO purged the political atmosphere of all warlike elements for a time, and left us in the long wished-for Halcyon repose of peace. But another Pharsalia will, ere long, arise in view; and nothing but our ARMS can preserve our independence. It is not a confederated Europe that Britain has to fear. It is an insidious poison flowing through her veins, administered in large pota-

---

* 1. The Army Medical Officer's Manual upon Active Service; or Precepts for his Guidance in the various Situations in which he may be placed, with Observations on the Preservation of the Health of Armies upon Foreign Service. By J. G. V. MILLINGEN, M. D. Surgeon to his Majesty's Forces, &c. One Volume, 8vo. pp. 268. London, 1819.

2. Practical Observations on the Means of preserving the Health of Soldiers in Camp and in Quarters; with Notes on the Medical Treatment of several of the most important Diseases which were found to prevail in the British Army during the late War. By EDWARD THORNHILL LUSCOMBE, M. D. Formerly Senior Surgeon of the 34th Regiment of Foot. One Volume, 8vo. pp. 132. Edinburgh, 1820.

tions of pseudo-philosophy, by the Atheists and Materialists of the day, that threatens the foundation of every institution, every principle, and every social covenant by which society has hitherto been held together! It is a positive fact, that nothing but the morality of the bayonet can now restrain the wickedness of the pen; and it is to our SOLDIERY that we must look for that liberty which our SOPHISTS are endeavouring to destroy.

To preserve the health, then, of our brave defenders, is an imperious duty and an important study; and the authors of the works before us have contributed their mites towards the construction of a code of Military Hygiene that may prove equally useful to the General and the Medical officer.

The substance of Dr. Luscombe's work, formed the Thesis which he defended at Edinburgh, in 1817, while graduating there, and the Senatus Academicus entertained so favourable an opinion of the production, that they expressed a wish to see it in print. It may be readily conceived that this wish was not very uncongenial to the wishes of the author; and accordingly, it has braved the ordeal of the press. Dr. Luscombe's rules of Hygiene appear to us sufficiently judicious, but add very little to the stock of knowledge already possessed by every intelligent army surgeon. The work, however, is principally intended for the *non-medical* officer's perusal; and there it will be very useful. The purely medical observations are very few, and do not profess to convey any novel or important information.

The work of Dr. Millingen is much more extensive, and practically useful, than that of Dr. Luscombe. Dr. M. enters into all the detail of Military Hygiene with a minuteness and perspicuity evincing the thorough knowledge which he has of his subject. The work is divided into twenty four Chapters, containing the following heads:—1. Organization of the Medical Department of an Expedition. 2. Formation of a Field Corps of Ambulance. 3. Medical Arrangements prior to an Expedition. 4. Medical Duties during a Voyage. 5. Disembarkation. 6. Establishment of general Hospitals. 7. Convalescent Hospitals. 8. Convalescent Depôts. 9. Officers' Hospital. 10. Hospital for Prisoners of War. 11. Preparations prior to a March. 12. Duties upon a March. 13. Bivouacs. 14. Encampments. 15. Cantonments. 16. Regimental Hospitals. 17. Intermediate Hospitals. 18. Mouvement of Sick and Wounded to the Rear. 19. Duties prior to a Battle. 20. Duties during a Battle. 21. Duties after a Battle. 22. Duties in a besieged Town. 23. Duties with a besieging Force. 24. Duties during a Retreat.

All these important subjects are discussed by Dr. Millengen with great talent, judgement, and practical good sense. The work should be in every army officer's library, whether medical or not; and to their attentive perusal and study, we cannot too strenuously recommend the book.

---

14. *Liquor Morphii Citratis.* From Dr. A. Oglevie Porter, of Bristol, we have the following Account and Formula of the Liquor Morphii Citratis, which we have no doubt will be gratefully received by his professional brethren.

Formula. ℞. Opii crudi optimi uncias quatuor; acidi citrici (cryst.) uncias duas; semel in mortario lapideo contunde; dein aquæ distillatæ bullientis octarium affunde, et intime misceantur; macera per horas viginti-quatuor; per chartam bibulosam cola; signetur preparatio — "*Liquor Morphii Citratis.*"

"It is desirable (says Dr. Porter) that the profession should be in legitimate possession of a preparation of opium that might supersede the temptation of having recourse to those which belong to the empire rather than the physician. Under this impression, I am desirous to promulgate a preparation not inferior to any yet discovered. I consider it equal in power to the Lancastrian Black Drop, as an anodyne; and equal to it, or Battley's "Sedative," in the peculiar medicinal excellencies of those preparations, in not materially exciting the circulation.

"The preparation was suggested to my mind, by having seen a saturated solution of opium in lime-juice used with great success in alleviating arthritic pain, about twenty-five years ago, in Spanish America. This recollection, with the light thrown upon the constituent principles of opium by the French Chemists, led me to try the preparation in question; and it has answered to my highest expectations. The formula I think is good: it is chemical, it is elegant, and the preparation will keep. I calculate its anodyne power at three times that of tincture of opium; yet it does not produce sensible excitement, and therefore its exhibition is seldom succeeded by heat, by head-ache, or by nausea. I have ventured to call the preparation 'Liquor Morphii Citratis,' a designation at once calculated to inform the physician of its nature, and to conceal it from the patient—points of no inconsiderable importance in practice."

Bristol, March 30, 1820.

